# Coronary thrombosis in a young male with COVID-19

**DOI:** 10.1016/j.idcr.2020.e00923

**Published:** 2020-07-25

**Authors:** Abdullah Shams, Fateen Ata, Kamran Mushtaq, Waqar Munir, Zohaib Yousaf

**Affiliations:** a Department of Internal Medicine, Hamad General Hospital, Hamad Medical Corporation, Doha, Qatar; b Department of Gastroenterology, Hamad General Hospital, Hamad Medical Corporation, Doha, Qatar; c Department of Infectious Disease, Communicable Disease Center, Hamad Medical Corporation, Doha, Qatar

**Keywords:** COVID-19, Myocardial infarction, Thrombosis, SARS-CoV-2

## Abstract

COVID-19 predominantly presents with respiratory symptoms, but other presentations are reported, including cardiac manifestations and thromboembolism. We present a healthy young gentleman with COVID-19 pneumonia, who developed acute ST-segment elevation myocardial infarction (STEMI) due to coronary thrombosis. He was managed successfully by primary percutaneous coronary intervention (PPCI).

## Highlights

• COVID-19 can present with a broad clinical spectrum of cardiac manifestations.
• The procoagulant state in COVID-19 can independently increase the risk of coronary thrombosis in otherwise healthy patients.
• COVID-19 patients who develop typical chest pain should be investigated for acute myocardial infarction.

## Case presentation

A 28-year-old gentleman of African origin, previously fit and well, presented with a three-day history of fever, dry cough, difficulty breathing, and myalgias. The patient had tachycardia (112 beats per minute), fever (39.2 degrees Celsius) tachypnea (30 breaths per minute) and desaturation (85 percent oxygen on room air) requiring 5 L oxygen via nasal cannula. However, he was normotensive. A physical examination, including the respiratory and cardiovascular system, was unremarkable.

Initial investigations revealed high inflammatory markers (CRP 92-normal range: 0–5 mg/L, ferritin 555-normal range: 38–270 u g/L, and lactic acid 3.5-normal range: 0.5–2.2 mmol/L). Sepsis workup was unrevealing apart from a positive SARS-CoV-2 nasopharyngeal real-time polymerase chain reaction (RT-PCR). Chest x-ray revealed prominent bilateral broncho-vascular markings with peripheral basal infiltrates in lower lung zones. A diagnosis of COVID-19 pneumonia of moderate severity was made. He received ceftriaxone (2 g intravenous daily), azithromycin (500 mg daily), and hydroxychloroquine (400 mg once daily) as per the local guidelines at the time. The patient responded well to the treatment, and by day eight was vitally stable, afebrile, and was maintaining saturation on room air.

**Fig. 1:**
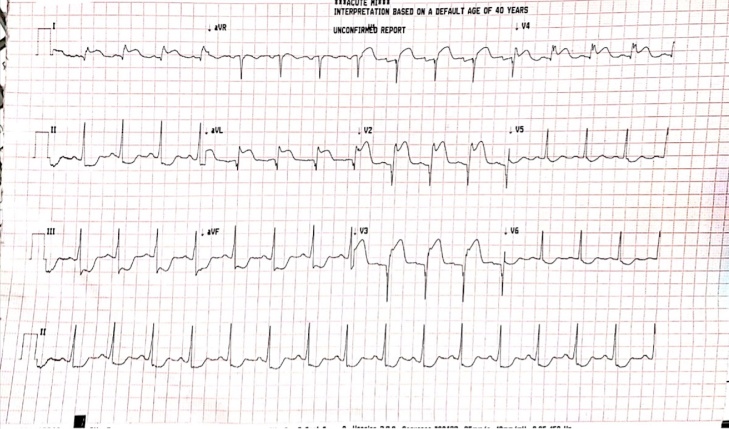
ECG ST elevation in the anterior leads (v1-v4.).

On day nine, the patient developed an acute severe, pressure like left-sided chest pain, radiating to his back. Physical exam, including the respiratory and cardiovascular system, was unremarkable, and he was vitally stable. An electrocardiogram (ECG) revealed ST-segment elevation in the anterior leads (v1-v4) (Fig. 1). Lab investigations revealed a rising trend of initial troponin-T level (first sample:148, after 8 h: 23821, 16 h:19209 - normal range 3−15 ng/L). A repeated chest x-ray did not show any new changes. An echocardiogram revealed low ejection fraction (EF) (28 %) with the dilated left ventricle and regional wall motion abnormalities. The basal anterior segment of the left ventricle was hypokinetic. The mid anterior, mid anteroseptal, mid-infero-septal, apical anterior, apical septal, apical inferior, apical lateral, and apex wall segments were akinetic.

**Fig. 2:**
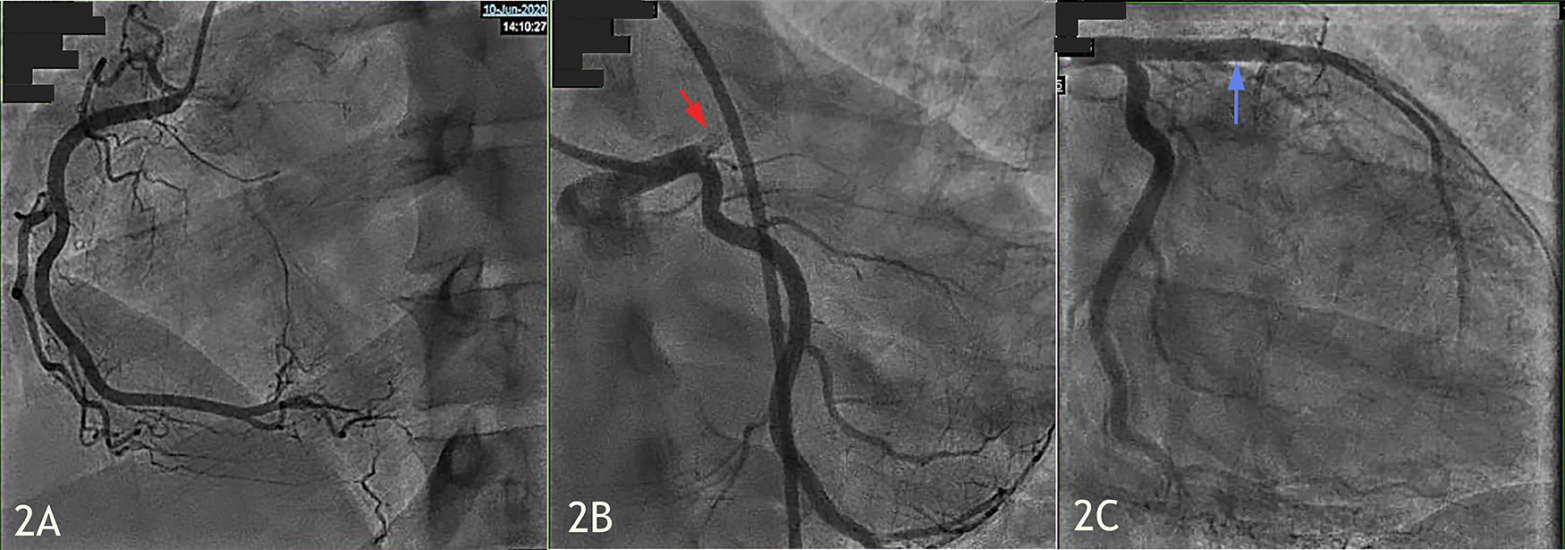
Coronary angiogram (2a. patent Right coronary artery (RCA), 2b. red arrow: thrombosed LAD, 2c. blue arrow: LAD post stenting).

With a diagnosis of an acute ST-elevation myocardial infarction, the patient was shifted to the nearest PPCI capable hospital within an hour. PPCI was performed as per the latest ACC guidelines for the management of STEMI in COVID-19 [1]. PPCI showed a thrombus in ostial-proximal left anterior descending (LAD) artery causing a 100 % stenosis and thrombolysis in myocardial infarction (TIMI) 0 flow (Fig. 2). Thrombus aspiration was performed, using a 6 F Export AP aspiration catheter. Pre-dilation was performed, using a Trek 2.5 × 15 mm compliant balloon. The inflation pressure was 8 ATM for 11.0 s. Drug-eluting stenting (DES) was performed, using a DES Xience Sierra 4.0 × 23 mm. The inflation pressure was 12 ATM for 15.0 s. Following the intervention, there was a 0 % residual stenosis, and TIMI 3 flow was achieved. Other coronary arteries were healthy.

Post-procedure, the patient received eptifibatide infusion for 18 h and heparin infusion for twenty-four hours. The patient received aspirin, clopidogrel, high-intensity atorvastatin, and metoprolol tartrate as per ACC guidelines. Cardiovascular risk factor screening for diabetes, dyslipidemia, and prothrombotic state (protein C, protein S, anticardiolipin antibody, and Factor V Leiden) were negative. The patient was started on beta-blockers and was planned to start angiotensin-converting enzyme inhibitors (ACE-i) later due to blood pressure on the lower side during hospitalization. Follow up in the cardiology clinic was arranged.

## Discussion

COVID-19 predominantly presents with respiratory symptoms such as fever, dry cough, myalgia, anorexia, and dyspnea. Gastrointestinal, neurological, and other atypical manifestations are also reported [2,3]. Cardiovascular manifestations are relatively rare; however, the acute coronary syndrome is reported [[4], [5], [6], [7]]. Our patient had no risk factors for coronary artery disease but developed an acute STEMI, and PPCI revealed complete thrombosis of LAD. An extensive workup for thromboembolism was negative. We believe that COVID-19 associated increased risk of thromboembolism was the most likely cause of LAD thrombus in this patient. The SARS-CoV-2 virus attaches to the angiotensin-converting enzyme receptor (ACE) with high affinity. ACE receptors are present on several tissues in the body, including the endothelium.

**Fig. 3:**
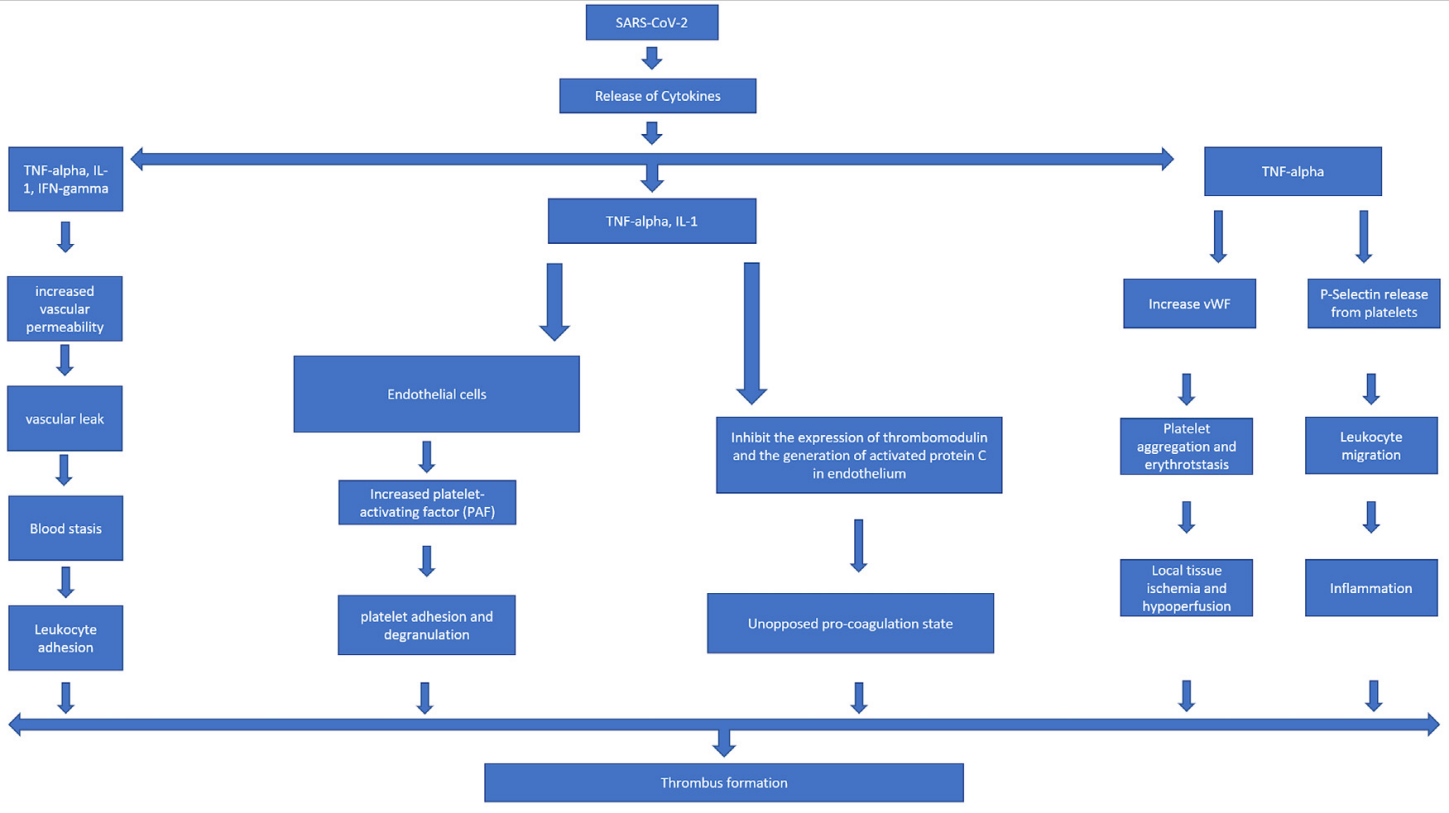
Proposed cytokine cascade in SARS-CoV-2 leading to thrombus formation.

The exact mechanism of coronary thrombus formation in COVID-19 is not known. Elevated inflammatory cytokines are present in patients with COVID-19 [8]. Pro-inflammatory cytokines activate the coagulation cascade and inhibit fibrinolysis. Tumor necrosis factor-alpha (TNF-α), interferon-gamma (IFN-γ) and interleukin 1 (IL-1) predominantly cause a procoagulant state in COVID-19. This procoagulant state can lead to leukocyte migration and adhesion, platelet activation and adhesion, and endothelial dysfunction resulting in thrombus formation. A proposed cascade of events leading to thrombosis in COVID-19 is shown in the flowchart (Fig. 3) [9].

In general, the management of STEMI in patients with COVID-19 is the same as in the pre-COVID era. It includes prompt recognition of symptoms, rapid laboratory analysis, including ECG, cardiac troponins, and primary percutaneous coronary intervention [1]. The main difference is related to a possible transfer delay secondary to COVID-19 for patients presenting to a non-PCI capable hospital. Patients presenting with STEMI and suspected COVID-19 in a PCI capable hospital should undergo PPCI. However, fibrinolysis is an alternative option for STEMI patients with suspected COVID presenting to non-PCI capable hospitals. In our case, prompt diagnosis and swift transfer to PPCI capable facility were made, and the patient had PPCI performed within 90 min of the onset of symptoms.

COVID-19 patients can present with acute coronary syndrome in patients with well-established risk factors [4].

There are reports of coronary thrombus in a young patient with risk factors for CAD and stent thrombosis in established CAD [5,10]. However, our patient was different as he was young with no conventional cardiovascular risk factors other than being a male. We believe this completes the clinical spectrum of several acute coronary syndrome presentations in patients with COVID-19.

## Funding

This work was financially supported by Qatar National Library.

## Consent

A written and signed consent was obtained from the patient prior to submission of the manuscript.

## Author contribution

Abdullah Shams: Literature review, case identification, case writing, final review, and approval of manuscript.

Fateen Ata: Initial manuscript writing and literature review, final review, and approval of manuscript.

Zohaib Yousaf: Manuscript writing, literature review, final review, and approval of manuscript.

Kamran Mushtaq: Manuscript writing, literature review, final review, and approval of manuscript.

## Declaration of Competing Interest

None of the authors have any conflict of interest to declare.
